# EB virus-induced ATR activation accelerates nasopharyngeal carcinoma growth via M2-type macrophages polarization

**DOI:** 10.1038/s41419-020-02925-9

**Published:** 2020-09-11

**Authors:** Bo Zhang, Tianyu Miao, Xin Shen, Lirong Bao, Cheng Zhang, Caixia Yan, Wei Wei, Jiao Chen, Liying Xiao, Chongkui Sun, Jintao Du, Yan Li

**Affiliations:** 1 State Key Laboratory of Oral Diseases, National Clinical Research Center for Oral Diseases, West China Hospital of Stomatology, Sichuan University, Chengdu, 610041 China; 2 Department of Stomatology, Minda Hospital of Hubei Minzu University, Enshi, 445000 China; 3 Vascular Surgery of West China Hospital, Sichuan University, Chengdu, 610041 China; 4 Otorhinolaryngology-Head and Neck Surgery of West China Hospital, Sichuan University, Chengdu, 610041 China

**Keywords:** Cancer microenvironment, Immunotherapy

## Abstract

Chronic inflammation induced by persistent viruses infection plays an essential role in tumor progression, which influenced on the interaction between the tumor cells and the tumor microenvironment. Our earlier study showed that ATR, a key kinase participant in single-stranded DNA damage response (DDR), was obviously activated by Epstein–Barr virus (EBV) in nasopharyngeal carcinoma (NPC). However, how EBV-induced ATR activation promotes NPC by influencing inflammatory microenvironment, such as tumor-associated macrophages (TAMs), remains elusive. In this study, we showed that EBV could promote the expression of p-ATR and M2-type TAMs transformation in clinical NPC specimens. The expression of p-ATR and M2-type TAMs were closely correlated each other and involved in TNM stage, lymph node metastasis and poor prognosis of the patients. In addition, the expression levels of CD68^+^CD206^+^, Arg1, VEGF, and CCL22 were increased in EB^+^ CNE1 cells, and decreased when ATR was inhibited. In the nude mice, EBV-induced ATR activation promoted subcutaneous transplanted tumor growth, higher expression of Ki67 and lung metastasis via M2-type TAMs recruitment. Experimental data also showed that the polarization of M2, the declined tumor necrosis factor-α (TNF-α) and increased transforming growth factor-β (TGF-β) were associated with ATR. Meanwhile, ATR activation could promote PPAR-δ and inhibited c-Jun and p-JNK expression, then downregulate JNK pathway. Collectively, our current study demonstrated the EBV infection could activate the ATR pathway to accelerate the transition of TAMs to M2, suggesting ATR knockdown could be a potential effective treatment strategy for EBV-positive NPC.

## Introduction

In recent years, it has been reported that 20% of the tumors are caused by microorganisms, and the virus play a great important role in tumorigenesis. After virus infection, the host cell genome become unstable, resulting in DNA damage responses (DDR) and gene mutation. The network of DDR is composed of multiple signal pathways, mainly including single-strand breaks (SSB) and double-strand breaks (DSB). Among them, DSB can mainly initiate ataxia telangiectasia mutation (ATM), while SSB can initiate ATM and Rad-3-related (ATR)^1^. Former studies have shown that EB virus infection activates DNA damage checkpoints by promoting the phosphorylation of ATM and checkpoint kinase 2 (CHK2)^2,3^. Meanwhile, our previous study has also revealed that^4^ Epstein–Barr virus (EBV) could activate the ATR-CHK1 pathway in nasopharyngeal carcinoma (NPC) cells, and ATR was a key factor in this process. Latent DNA virus infection related tumorigenesis are closely related with that virus proteins interfere with DDR, causing host cell genome mutation accumulation and DNA fragmentation, thus change other signaling pathways to promote the tumor development^5,6^.

Tumor-associated macrophages (TAMs) are the most abundant recruited inflammatory cells by virus in the tumor microenvironment (TME), which are closely associated with poor prognosis in various tumor types^7–10^. TAMs are affected by interferon regulatory factors and transcriptional activators, and thus exhibit phenotypes with different functions, the most important ones of which are M1 (classical activation) and M2 (selective activation). M1 produces a pro-inflammatory Th1 immune response and exerts tumoricidal activity in the early stages of cancer^11^, while M2 is associated with an anti-inflammatory Th2 immune response and plays a role in promoting cancer in the late stage of the tumor^12^. Therefore, TAMs were thought to have the dual regulating effects. Previous studies have shown that^13,14^, Hepatitis B Virus (HBV) and human papillomavirus (HPV)-infected tumor cells can secrete a large number of chemokines and cytokines (such as IL-4, IL-2, IL-10, etc.), and promote the transformation of macrophages to M2, hinting that viral infection might play an important role in the macrophage M2-type polarization. Meanwhile, viral infection could also regulate the polarization of TAMs via inducing DDR^15–17^. For example, ATR mutations lead to the decreased T cell recruitment, stimulate the increase in the number of M2 macrophages associated with tumor invasion and promote melanoma growth^18^, indicating that endogenous ATR mutations results in M2 TAMs accumulation.

However, whether EBV infection affects the polarization of TAMs and its specific mechanisms are still undiscovered, and it is still not clear how EBV-driven ATR activation interacts with TAMs recruitment, which is involved in genome stability and immune regulation. In this study, the effects of ATR on TAMs polarization were investigated in EBV-positive NPC cells in vitro and in vivo. The underlying mechanisms of EBV-induced ATR on TAMs polarization were demonstrated, which were associated with TGF-β, PPAR-δ, and JNK pathway.

## Results

### EBV infection-induced ATR activation and M2-type TAMs transformation in clinical NPC specimens

**Fig. 1:**
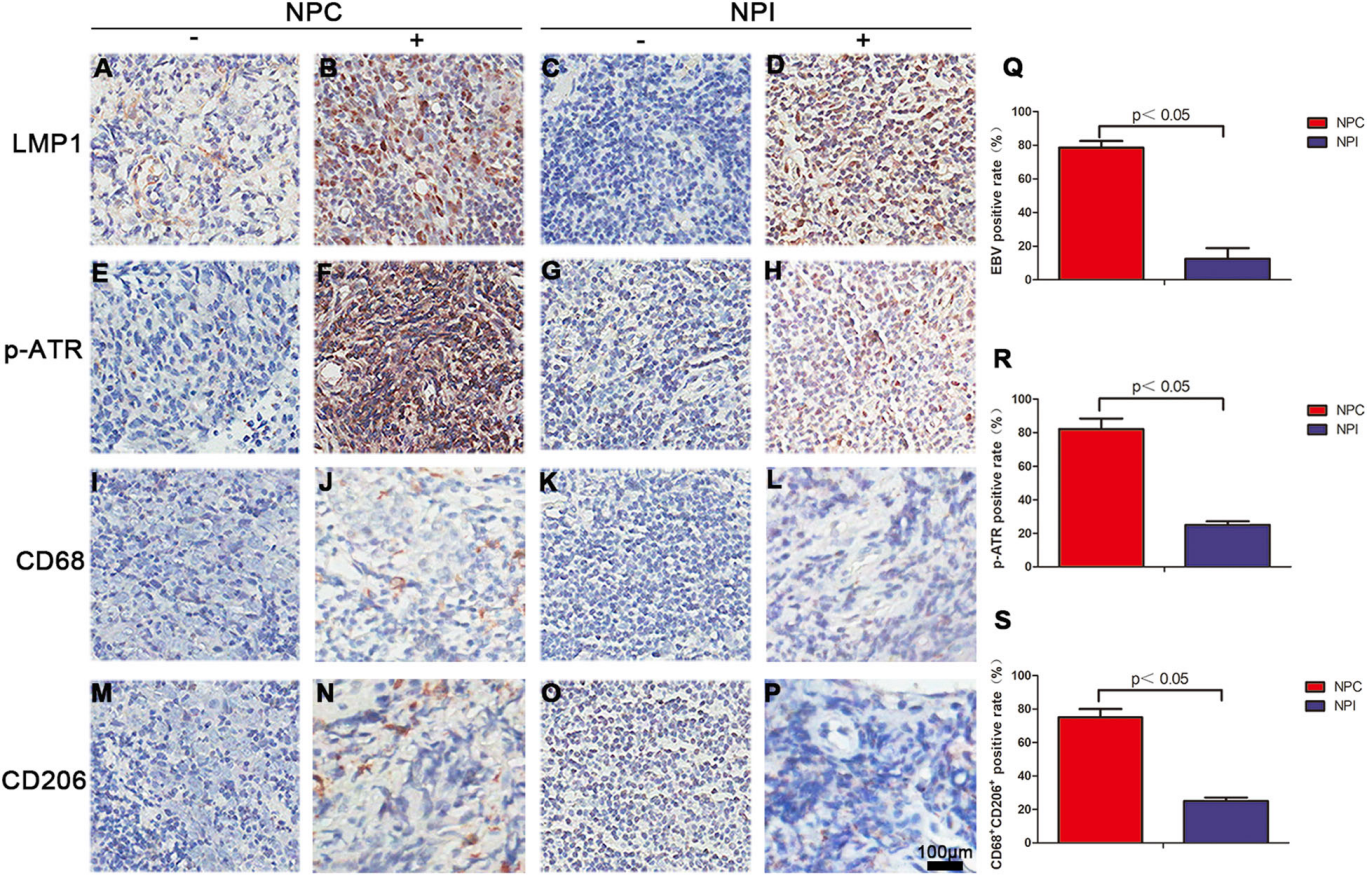
LMP1, p-ATR, CD68, and CD206 expressions in clinical samples of NPC and NPI. **a**–**p** Representative IHC results for LMP1, p-ATR, CD68, and CD206 (**a**, **e**, **i**, **m** negative; **b**, **f**, **j**, **n** positive) in human NPC samples or (**c**, **g**, **k**, **o** negative; **d**, **h**, **l**, **p** positive) in human NPI samples. **q** EBV-positive rate detected by IHC in NPC and NPI samples. **r** Percentage of p-ATR-positive specimens in NPC and NPI samples. **s** Percentage of CD68^+^CD206^+^-positive specimens in NPC and NPI samples.

To determine whether EBV infection elevated the level of p-ATR and CD68^+^/CD206^+^, immunohistochemistry was performed in 28 NPC and 24 NPI specimens. EBV infection marker LMP1, mainly expressed in the cell membrane or cytoplasm, is visible as brown yellow particles or plaque (Fig. 1b), while the negative expression is undetectable (Fig. 1a), In NPI, the negative and positive expression were shown in Fig. 1c, d, respectively. The data shows 78.6% (22/28) of the NPC specimens are EBV positive, while it is only 12.5% (3/24) in NPI. Therefore, the EBV infection was significantly higher in the NPC group than in the NPI group (*p* < 0.05; Fig. 1q). As far as p-ATR regarded, brown particles were observed mainly in the cytoplasm and nucleus (Fig. 1e–h). The positive rate in NPC samples was 82.1% (23/28, Fig. 1r). Distribution of M2 (CD68/CD206 positive expression) showed typical macrophage morphology with brown-stained areas in the cell membrane and cytoplasm in NPC and NPI (Fig. 1i–p) samples. The positive rates of M2 in NPC was 75% (21/28), far greater than that in NPI samples (Fig. 1s).

In addition, the relationship between the expression levels of p-ATR, CD68/CD206 and EBV infection in NPC specimens was evaluated respectively. The p-ATR and CD68^+^/CD206^+^ levels were observed as 95.5% (21/22) and 81.8% (18/22) in the EBV-positive NPC biopsies, respectively, while only 33.3% and 50.0% in the EBV-negative NPC, respectively (Table 1, *p < 0.05). The levels of LMP1, p-ATR, and CD68^+^/CD206^+^ in NPC were not correlated with age and sex, but positively correlated with TNM stage and lymph node metastasis rate, which indicated a poor prognosis (Table 2, *p < 0.05). The location of p-ATR and CD68 was simultaneously detected by fluorescent staining (SFig. 1).

**Table 1:** Correlation analysis of EBV and p-ATR, CD68^+^CD206^+^ detection rates in NPC clinical specimens.

Group	Number	p-ATR		*p* value	CD68^+^CD206^+^		*p* value
		Positive(%)	Negative (%)		Positive (%)	Negative (%)	
EBV-positive	22	21 (95.5)	1 (4.5)	<0.05	18 (81.8)	4 (18.2)	<0.05
EBV-negative	6	2 (33.3)	4 (66.7)		3 (50.0)	3 (50.0)

**Table 2:** Relationship between EBV infection, p-ATR and CD68^+^CD206^+^ positive expression and epidemiological indicators in NPC tissues.

Epidemiological index	The number of cases	EB infection		p-ATR		CD68^+^CD206^+^		*p* values
		Positive (%)	Negative (%)	Positive (%)	Negative (%)	Positive (%)	Negative (%)	
Age								>0.05
≤40	3	2 (66.7)	1 (33.3)	3 (100.0)	0 (0.0)	2 (66.7)	1 (33.3)	
40–60	13	10 (76.9)	3 (23.1)	11 (84.6)	2 (15.4)	8 (61.5)	5 (38.5)	
>60	12	10 (83.3)	2 (16.7)	9 (75.0)	3 (25.0)	11 (91.7)	1 (8.3)	
Gender								>0.05
Male	19	14 (73.7)	5 (26.3)	17 (89.5)	2 (10.5)	15 (78.9)	4 (21.1)	
Female	9	8 (88.9)	1 (11.1)	6 (66.7)	3 (33.3)	6 (66.7)	3 (33.3)	
TNM								<0.05
I–II	14	9 (64.3)	5 (35.7)	9 (64.3)	5 (35.7)	8 (57.1)	6 (42.9)	
III–IV	14	13 (92.9)	1 (7.1)	14 (100.0)	0 (0.0)	13 (92.9)	1 (7.1)	
LNM								<0.05
+	21	20 (95.2)	1 (4.8)	20 (95.2)	1 (4.8)	17 (81.0)	4 (19.0)	
−	7	2 (28.6)	5 (71.4)	3 (42.9)	4 (57.1)	4 (57.1)	3 (42.9)	

*TNM* tumor node metastasis, *LNM* lymph node metastasis.

### ATR activated M2 TAMs polarization after EB virus infection in vitro

As for the tumor cells-macrophages co-culture system establishment, EB^+^shATR and EB^+^shNC CNE1 cells were identified as shown as SFig. 2. At the same time, THP-1 cells were induced into non-polarized macrophages (M0) successfully and analyzed by flow cytometry analysis (FCM) (SFig. 3A, B).

**Fig. 2:**
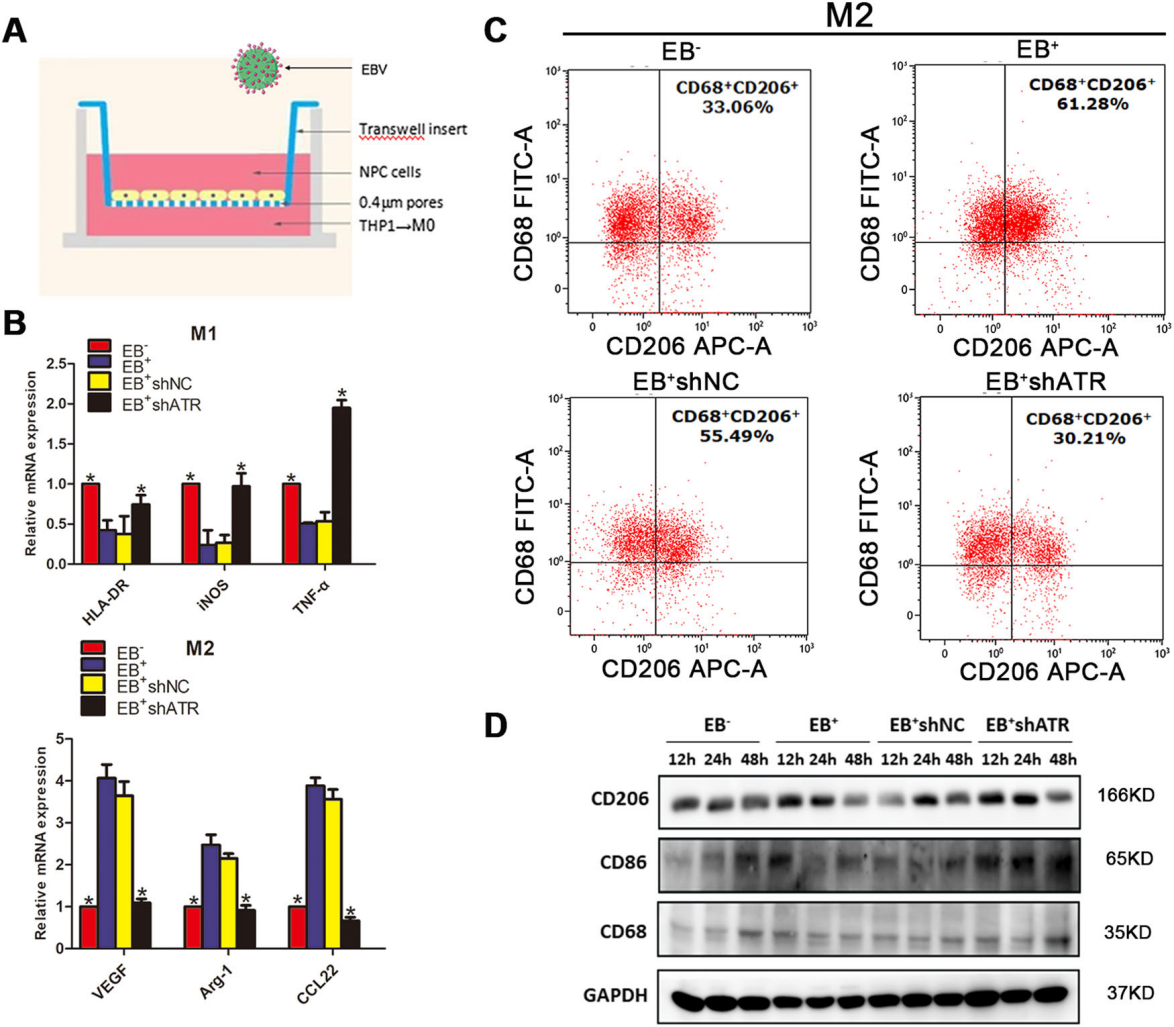
ATR-promoted M2 polarization in EB^+^CNE1 cells. **a** Schematic model of the co-culture system of macrophages and NPC cells; EB^+^CNE1 cells stably expressing shNC or shATR were co-cultured with M0 macrophages for 2 days, and EB^−^ and EB^+^ were used as negative and positive controls, respectively. **b** qPCR determined mRNA levels of M1 and M2 macrophage markers; **c** Detection of the expression of CD68^+^/CD206^+^ by FCM. **d** WB detection of protein expression of CD68, CD86, and CD206 at different time points after co-culture. Data are represented as the mean ± SD of three independent experiments. *p < 0.05.

From co-culture experiments in Fig. 2a, the mRNA and protein expression levels of M1 and M2 were detected in each group. The mRNA expression of M1 markers (HLA-DR, iNOS, and TNF-α) were increased obviously, while the M2 markers (Arg1, VEGF, and CCL22) downregulated significantly in the EB^−^ and EB^+^shATR groups compared with the EB^+^ and EB^+^shNC groups (*p < 0.05, Fig. 2b).

EBV infection promoted M2 TAMs polarization from 33.06% to 61.28% compared with the EB^−^ group, resulting in an increase in CD68^+^/CD206^+^ expression. When ATR being interfered, the ratio of CD68^+^/CD206^+^ decreased significantly from 55.49% to 30.21% compared with the shNC group (Fig. 2c).

The results of WB showed that the expression of CD68 in the EB^−^ group gradually increased with time, but there was no significant difference between the EB^+^, EB^+^shNC and EB^+^shATR group from 12 to 48 h. Meanwhile, CD206 in EB^+^ and EB^+^shNC group increased sharply between 12 and 24 h, while CD86 in EB^−^ and EB^+^shATR group increased gradually (Fig. 2d). We treated EB^−^ and EB^+^ group with 5 μM AZD0156 (ATM inhibitor) for 24 h, respectively. The WB results showed no significant difference in the expression of CD68, while CD86 and CD206 were slightly reduced (SFig. 4).

### In nude mice xenografts, ATR activated by EBV enhanced tumor development and M2 polarization

**Fig. 3:**
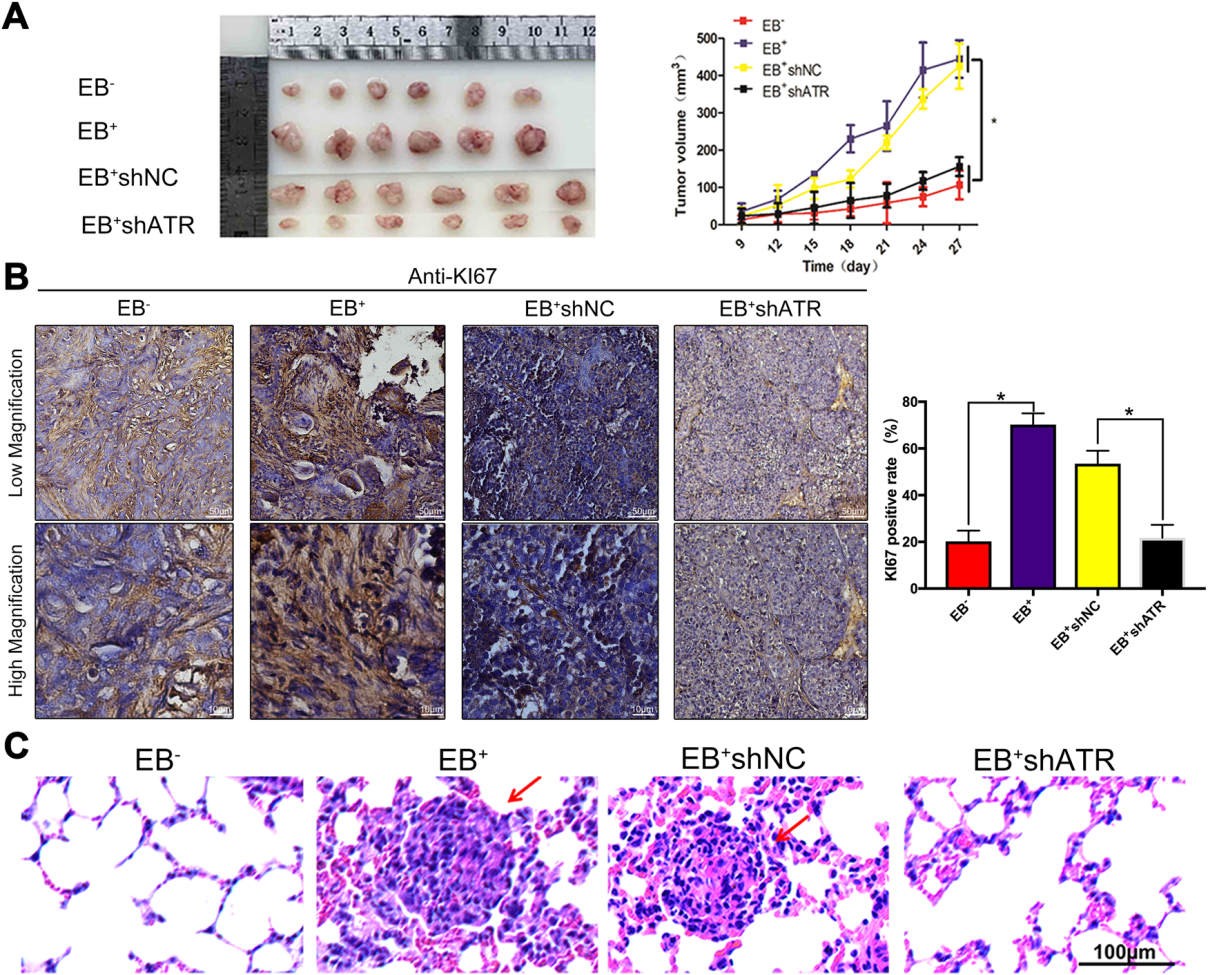
ATR activated by EBV promoted NPC xenograft growth and lung metastasis. **a** The tumors from the 11th day were measured and the volume of tumors were calculated *p < 0.05; **b** Representative IHC results for Ki67 in nude mice xenografts tumor samples and the percentage of Ki67-positive cells in nude mice xenografts tumor samples. *p < 0.05. **c** HE staining of the lungs in nude mice of NPC tumor-bearing mice.

**Fig. 4:**
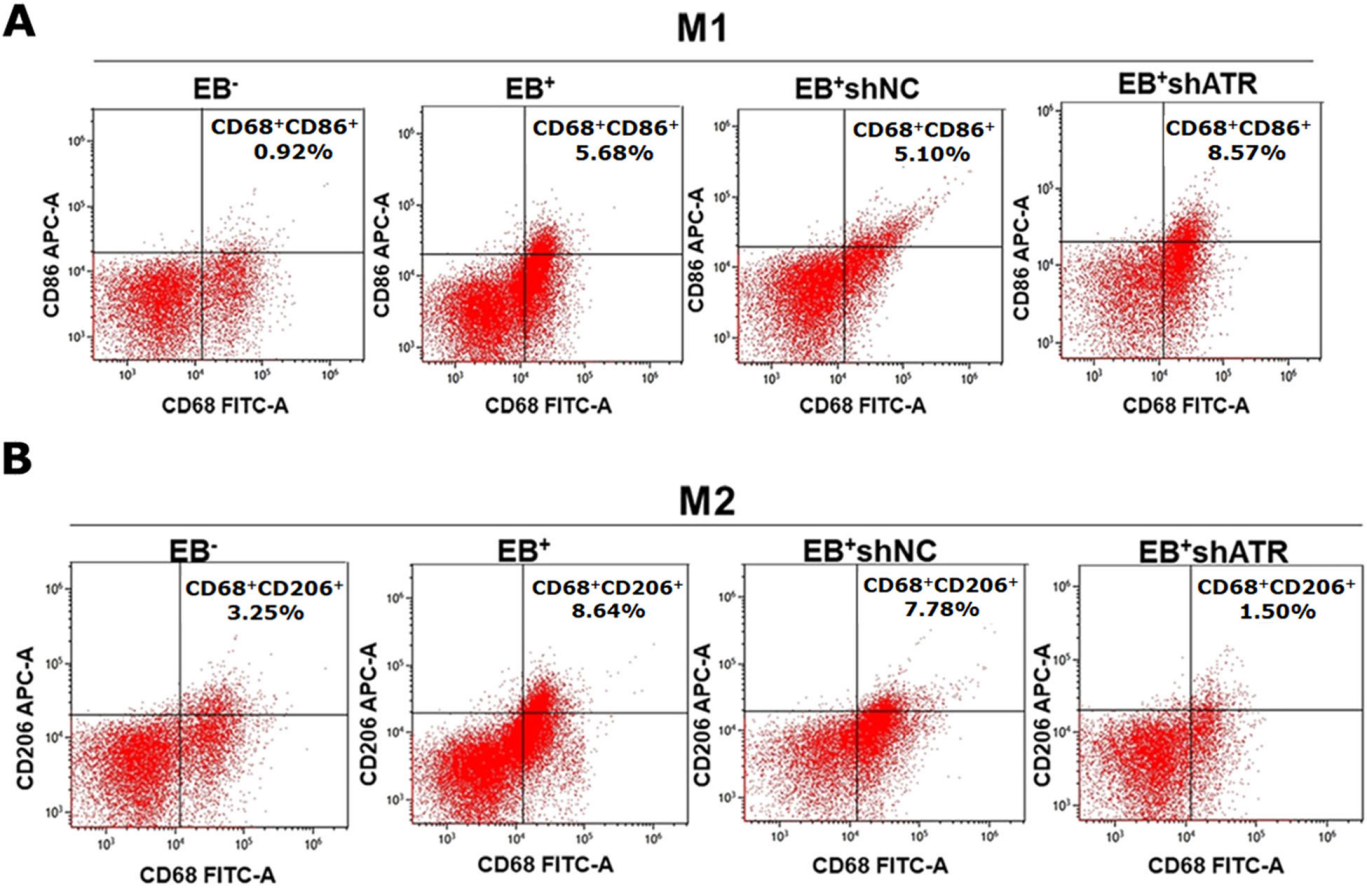
EBV promoted M2 TAMs polarization in NPC xenografts, which was reversed by shATR. **a** Expression of M1 (CD68^+^CD86^+^) TAMs in nude mice xenografts by FCM; **b** Expression of M2 (CD68^+^CD206^+^) TAMs in nude mice xenografts by FCM.

The tumor growth in the EB^+^ and EB^+^shNC groups was rapid with the large tumor volumes of 444.2651 ± 50.447 mm^3^ and 425.0146 ± 60.338 mm^3^, respectively at 27 days after injection subcutaneously. But the tumor growth in EB^−^ and EB^+^shATR groups was obviously delayed with the tumor volumes of 106.5484 ± 38.383 mm^3^ and 155.9888 ± 25.178 mm^3^, respectively, which were just about 1/4–1/3 of the EB^+^ group (*p < 0.05, Fig. 3a). In the group of EB^+^ and EB^+^shNC, the morphology of nucleus was megakaryocytes, binuclear or polynuclear. A brownish black nucleus indicates positive Ki67 expression. Consistent with the phenotypic trend of the organization, the results of IHC showed that the expression of Ki67 was higher in tumor tissues of EB^+^ group and EB^+^shNC significantly, while it was lesser expressed in EB^+^shATR group (*p < 0.05, Fig. 3b). The armpits subcutaneous transplanted tumors in the EB^+^ group and the EB^+^shNC group resulted in the lung metastasis (shown by red arrows in Fig. 3c). The incidence of pulmonary metastases was 16.7% (1/6) in both two groups, but no pulmonary metastasis was observed in the EB^−^ and EB^+^shATR tumor models. FCM detected M1 (CD68^+^/CD86^+^) and M2 (CD68^+^/CD206^+^) markers in four groups of nude mice xenografts (Fig. 4a, b). Compared to 5.68% for M1 in EB^+^ group, the proportion was about 8.57% for M1 in EB^+^shATR tumor tissues, which was higher. Meanwhile, the proportion of M2 in EB^+^shATR group was about 1.50%, which was significantly lower than 8.64% for M2 in EB^+^ group.

### M2 polarization by EBV-driven ATR activation might be involved in TGF-β secretion and JNK pathway inhibition

According to the literature report^19^, nine TAMs polarization factors were screened as listed in the SFig. 5A. The correlation between these factors and ATR was detected in NSCC by Timer software. From the cor values, it was found that CCL17, TNF, and TGF-β1 were highly correlated with ATR (corå 0.1, *p* < 0.05, SFig. 5B). Then the latter two were chosen for ELISA verification.

**Fig. 5:**
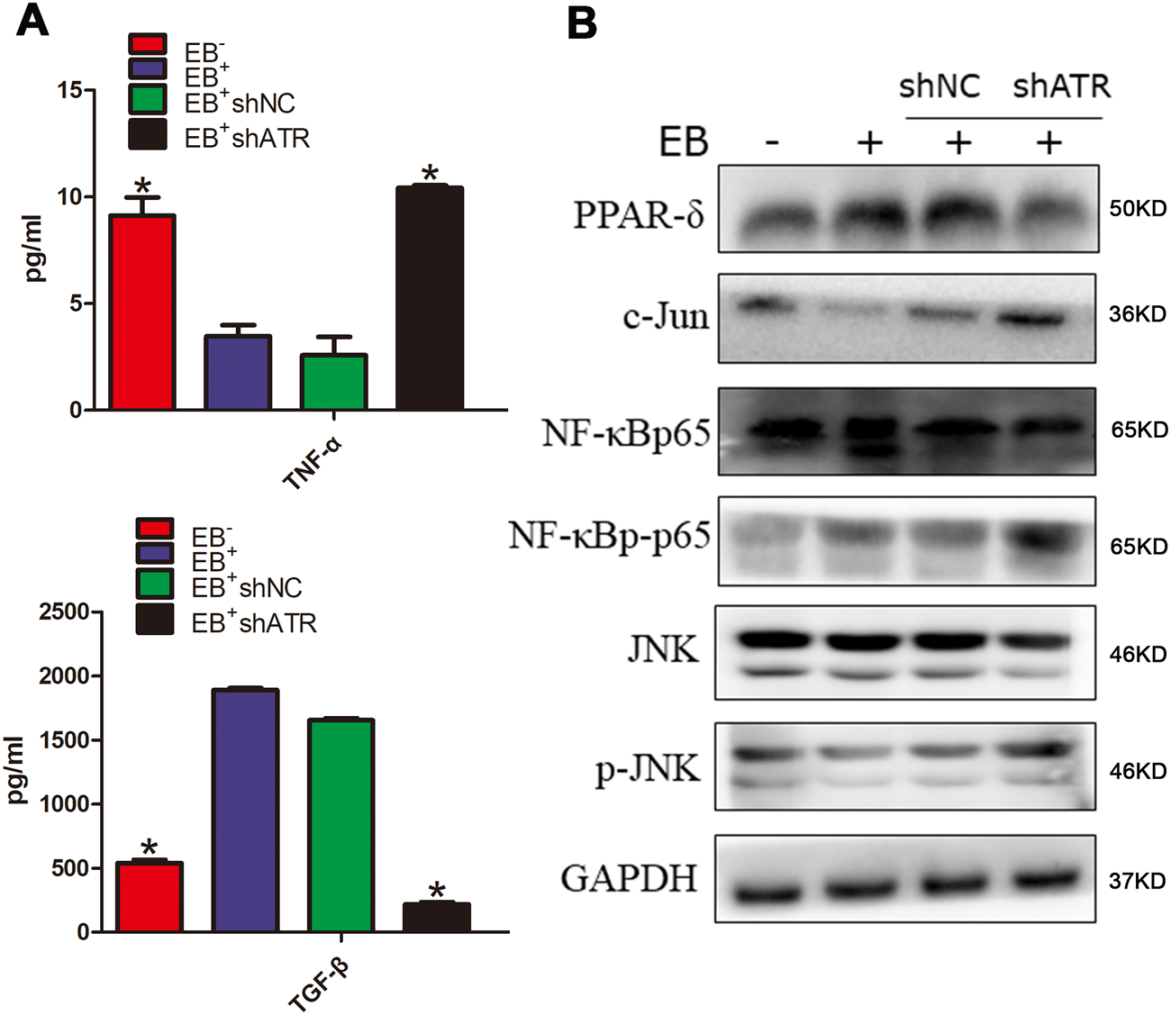
EBV-induced ATR promoted the TGF-β secretion and inhibited the JNK pathway activation, which could reversed by shATR. **a** Detection of TNF-α and TGF-β in cultured supernatant by ELISA. Data are represented as the mean ± SD of three independent experiments. *p < 0.05. **b** WB detection of protein expression levels of PPAR-δ, NF-κBp65, NF-κBp-p65, c-Jun, JNK, and p-JNK.

TNF-α and TGF-β levels in the culture supernatant were determined by ELISA. The results showed that TNF-α in EB^+^ group and EB^+^shNC group was significantly lower than that in EB^-^ group and EB^+^shATR group. However, the level of TGF-β was in the opposite (Fig. 5a).

The results of WB showed that, compared with EB^−^ group, EBV infection could promote the expression of PPAR-δ and NF-κBp-p65, and inhibit the expression of c-Jun and JNK phosphorylation. Compared with EB^+^shNC group, intervention ATR could promote c-Jun expression and JNK phosphorylation, accompanied by the expression inhibition of PPAR-δ. EBV infection and ATR interference exhibited no obvious effect on the expression of total NF-κBp65 and JNK proteins (Fig. 5b).

**Fig. 6:**
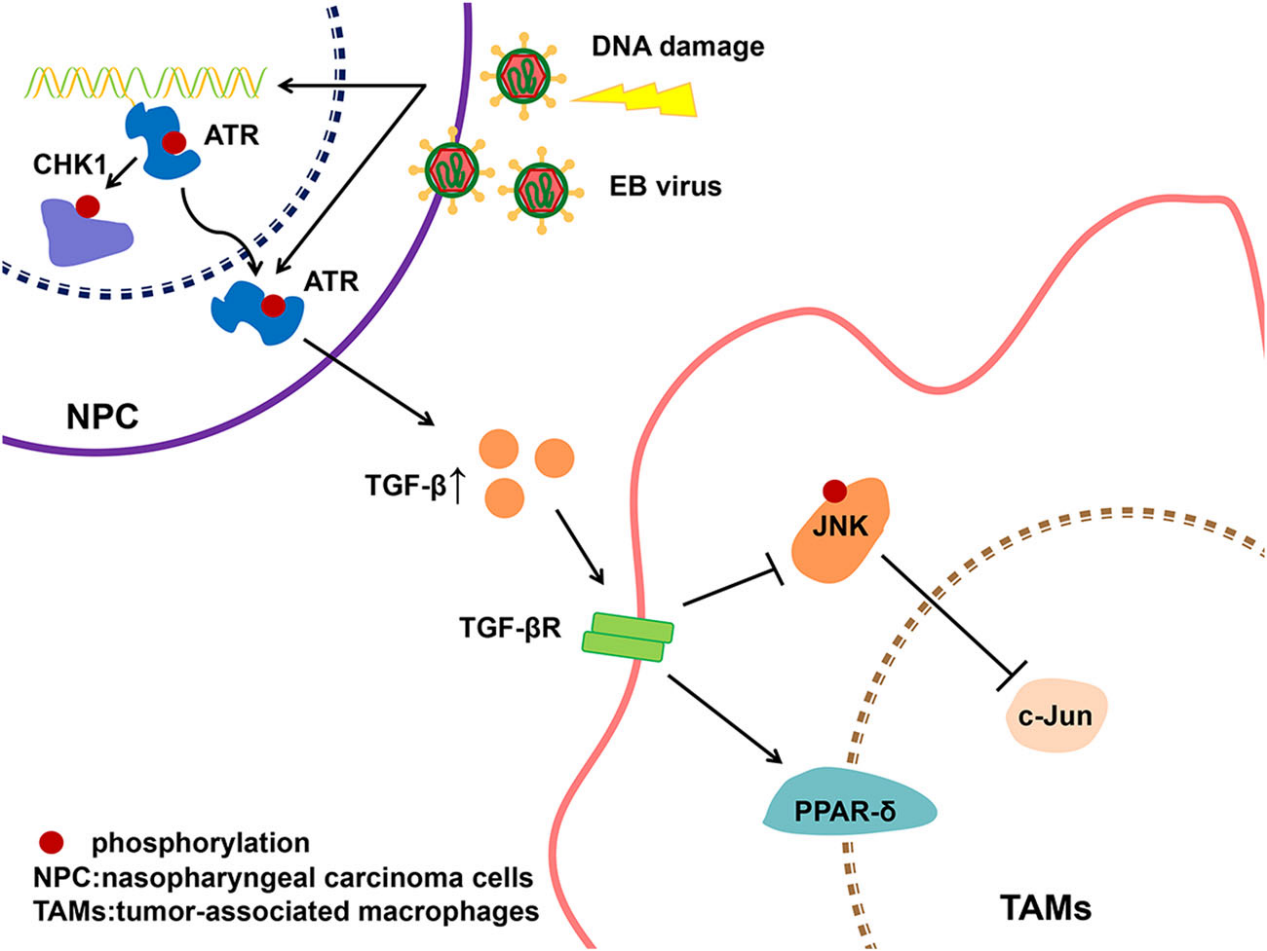
Model for EBV-mediated ATR promoted the polarization of M2 TAMs. EBV activated the JNK pathway by activating ATR, and simultaneously increased TGF-β and decreased TNF-α, and promoted the polarization of M2 TAMs with PPAR-δ as an activating factor. The gene interfered with the expression of ATR, which resulted in the conversion of M2 to M1 TAMs with c-Jun as an activating factor.

Our findings indicated that EB virus could promote M2 polarization of TAMs, and ATR activation induced by EBV might play a major role, in which TGF-β and PPAR-δ increased contrary with JNK pathway (including c-Jun and p-JNK) decreased. ATR interference could reverse the transformation of M2 to M1 TAMs. Those results support a model in which EBV-mediated ATR promotes polarization of M2 TAMs. Therefore, ATR regulation might represent a promising strategy for EBV-positive NPC (Fig. 6).

## Discussion

The development of tumors is not only determined by the tumor cells themselves but also closely related to various immunosuppressive cells in the surrounding TME^20^. Among them, TAMs account for the largest proportion and plasticity and divide M1 and M2 phenotypes due to the surface molecular difference. It is generally believed that TAMs in tumors are mainly M2-type with CD68^+^/CD206^+^ specific marker^21–25^, which can promote the invasion and metastasis of tumors and affect the treatment and prognosis of patients. Caro et al.^26^ have detected the expression of CD68 and CD206 in 110 cases of pancreatic cancer by IHC, indicating that the positive proportion of M2 (CD68^+^CD206^+^) TAMs is 63%. In hepatocellular carcinoma, the expression of M2 TAMs by IHC has been also detected, suggesting that the positive proportion reaches 68%^27^. It has also been found that M2-type TAMs infiltration accounts for 75% of NPC tissues, which is consistent with previous research^28^.

The virus play an important role in promoting the polarization of M2 TAMs. HBV-induced immune damage promotes the polarization of M2 macrophages^29,30^. HPV detected in the rectal squamous cell carcinoma is associated with the increased macrophage M2 polarization^31^. In addition, the swine influenza virus^32^ and rhinovirus^33^ can also be involved in the regulation of macrophage conversion from M1 to M2 polarization. Saha et al.^34^ also found that hepatitis C virus can induce monocytes to differentiate into polarized M2 macrophages, which promotes the activation of stellate cells by TGF-β. Their E2 envelope protein enhances Stat-3 and inhibits the activation of Stat1, promoting macrophage polarization to the M2 phenotype^35^. Currently, there is controversy about the polarizing effect of EBV on TAMs in NPC. In a recent study, M2 TAMs are more invasive in clinical specimens of EBV-negative NPC^36^. However, Huang^37^ and our group stained NPC tissues with the EBV infection marker and the M2 marker, indicating that the positive expression rate of M2 TAMs is highly correlated with the EBV infection. The reason why our results are different may be due to the presence of different M2 subtypes with other cytokine profiles between EBV-negative and positive NPC. As has been reported, EBV-positive NPC has a characteristic serological profile with antibodies against the EBV viral capsid antigen and early antigen IgA^38^, which could induce M2b polarization. And as there is a possible recruitment problem of regulatory T cells (Tregs) in EBV-negative NPC, the most likely TAMs in this subgroup would then be the M2c macrophages^36^. In order to determine whether EBV can promote M2 TAMs polarization in NPC cells, according to the method^39^, a transwell chamber noncontact co-culture system was designed to mimic the interaction of macrophages with CNE1 cells. The results showed that EBV promoted the polarization of M2 TAMs in NPC cells, such as the enhancement of M2 inducer mRNA (Arg1, VEGF, and CCL22) and upregulation of M2-type marker (CD68^+^CD206^+^).

TAMs invasion is associated with tumor stage and lymph node metastasis, which is a strong correlation between the invasion density and poor prognosis of various types of human cancer^40,41^. On one hand, high expression of M2-macrophages molecules is thought to enhance tumor metastasis and angiogenic ability to promote tumor development. On the other hand, M2 plays an immunosuppressive role in inhibiting the antitumor immunity of M1 and Th1 cells which involved in the recruiting of regulatory T cells and Th2 cells^25,42^. Our results showed that the expression of p-ATR and CD68^+^CD206^+^ in EB virus-positive NPC was not correlated with age and sex, but with TNM stage and lymph node metastasis, suggesting that the high level of p-ATR and M2 is related to the poor prognosis of the patients. Moreover, the tumor transplantation experiment in nude mice also revealed that the higher M2 macrophages promote the lung metastasis of the subcutaneous tumors.

There is a mutual action between DDR and TME (mainly TAMs). DDR regulates TME secreted protease and related cytokines to influence the progression of NPC, for example, IL-6, IL-8, IL-27, EGF, metalloproteinase, and WNT family members^15^. DDR can also lead to the production of cancer-related inflammation, which induces TME-secreting cytokines such as TNF, IL-1, IL-6, and chemokines such as CCL2 and CXCL8, activates transcription factors of NF-κB and Stat-3, and promotes tumor proliferation, invasion, metastasis, and treatment resistance^16,43^. Meanwhile, TME can also regulate DDR. A previous study^44^ pointed out that TGF-β is the most secreted cytokine in TME, which can control the DDR pathway by regulating ATM-CHK2. More specifically, some studies have reported that there is a link between ATR and TAMs. For example, the viral protein R (Vpr) is an auxiliary gene of HIV. The early in vivo studies have demonstrated that the HIV-1 infection of human primary CD4 lymphocytes leads to G2 arrest in a Vpr-dependent manner, which requires the progress of ATR-mediated^45^. Vpr induces a change in DNA structure to initiate an ATR-mediated DDR, leading to a viral infection of macrophages^46^, suggesting that the viral-mediated ATR pathway will have an impact on macrophages. In addition, ATR mutation of the melanoma can induce the accumulation of M2 TAMs and promote tumor proliferation, indicating that ATR mutation may be related to the polarization of M2 TAMs^18^. But no direct evidence can support the relationship between ATR and M2. Our data have shown that EBV can activate the ATR-mediated DDR pathway by phosphorylating ATR-CHK1 in NPC cells, and ATR is a key factor in this pathway. Correlation analysis in clinical subjects and animal experiments showed that there was a certain correlation between the positive expression of p-ATR and M2 TAMs in EBV-positive NPC tissues.

Furthermore, M2 polarization factors involved in ATR were investigated. Cytokines and chemokines play important roles in the polarization of macrophages. Common factors involved in polarization are IL-4, IL-13, IL-6, IL-10, IL-12, TNF, TGF-β, CCL22, CCL17, CCL2, etc^19^. TIMER^47^ is a comprehensive resource for systematic analysis of immune infiltration of different cancer types, which can assess six immune infiltrates (B cells, CD4^+^ T cells, CD8^+^ T cells, neutrophils, macrophages, and abundance of dendritic cells). To better understand the correlation between ATR and pro-macrophage polarization factors in HNSCC, TIMER software was used to detect and evaluate the cor value, and it was found that the macrophage polarization factors TNF and TGF-β1 may be related with ATR. TNF-α is a common inhibitor of M2 TAMs, which can inhibit the differentiation of macrophages to M2 by direct or indirect production of IL-13, thereby reducing the number of M2 TAMs^48,49^. TGF-β promotes the differentiation of nonactivated macrophages into M2 TAMs by upregulating the expression of IL-10 and downregulating the expression of TNF-α and IL-12^50,51^. The expression of TNF-α and TGF-β in co-culture supernatants of macrophages and NPC cells was detected by ELISA. The results indicated that interference ATR could reverse this effect, resulting in a decrease of TGF-β and the increase of TNF-α.

Monocytes/macrophages can undergo a dynamic conversion of their phenotype/function in response to the signals coming from ATR. c-Jun and PPAR-δ are the activators for the interconversion of M1 or M2 macrophages. In the study of obesity-induced hepatic inflammation, the increase of pro-inflammatory cytokines, and the acute phase reactants, activation of NF-κB and JNK pathways can increase the expression of c-Jun, thereby promoting M1-type polarization. Conversely, inhibition of the JNK pathway proceeds to the M2-type polarization^52,53^. Moreover, one study has shown that PPAR-δ is activated in DDR and acts as an immunosuppressive agent by inhibiting the production of pro-inflammatory cytokines^54^. We demonstrated that EBV promoted the macrophage PPAR-δ and NF-κBp-p65 expression and decreased c-Jun and p-JNK expression. Interfering with ATR-inhibited PPAR-δ and promoted c-Jun and p-JNK expression, suggesting EBV-induced M2-macrophages polarization and its mechanism might be ATR-inhibited JNK pathway, rather than the NF-κB pathway.

This study investigated the role of ATR on TAMs polarization in EBV-positive NPC, and the regulation mechanism of tumor genomic stability on the TME. It is expected to develop a new immune approach to treat EB^+^ NPC.

## Materials and methods

### Collection of NPC and nasopharyngeal inflammation (NPI) clinical specimens

A total of 28 paraformaldehyde-fixed NPC tissues with histopathology reports were collected. The average age of the patients was 57 ± 15 years. Twenty-four patients with NPI (average age: 42 ± 17 years) were selected as the controls. All 52 tissue specimens were procured from the Minda Hospital of Hubei Minzu University and the West China Hospital of Sichuan University from 2017 to 2020. The study was carried out under the approval and supervision of the Ethics Committee (K2017018 and WCHSIRB-D-2020-206), and conducted in accordance with the Declaration of Helsinki. The experiments were performed in accordance with approved guidelines. The written informed consents were signed for each patient.

### Immunohistochemistry

The 52 clinical specimens and the tumors of the mice were analyzed by immunohistochemical method^55,56^. In brief, 4 μm formalin-fixed and paraffin-embedded sections were de-paraffinized with xylene twice and rehydrated in graded 100%, 90%, 80%, and 70% alcohol solution, and the antigens were retrieved with Tris-EDTA buffer for 3–5 min at 100 °C. The slides were peroxidase blocked with 3% hydrogen peroxide solution for 10 min and then blocked using 5% bovine serum albumin (Sigma) for 30 min. Slides were incubated with a primary antibody against latent membrane protein 1 (LMP1) (1:100; ab182153, Abcam), phosphorylated-ATR (1:100; ab178407, Abcam), CD68 (0.5 μg/ml; ab125212, Abcam), CD206 (0.5 μg/ml; ab64693, Abcam), and Ki67 (1:200; YM3064, Immunoway) for overnight. On the secondary day, the slides were detected by the ChemMate EnVision kit (Dako, Carpinteria, CA, USA). Immune reactivity was analyzed and quantified using ImageScope software (Aperio Technology, Vista, CA, USA). The investigator was blinded to the group allocation during the experiment.

### Cell culture, EB virus infection, RNAi lentivirus infection, EB^+^CNE1 and M0 macrophages co-culture system establishment

CNE1 (an EBV-negative NPC cell line), B95-8 (a marmoset EBV-immortalized B cell line), and THP-1 (a human mononuclear macrophage cell line) were friendly given by Li Shengfu, Zhang Zairong, and Li Jing came from Sichuan University, respectively. The cells were cultured in RPMI-1640 (HyClone, Logan, UT, USA) with 10% FBS (Gibco, Grand Island, NY, USA) and 1% penicillin-streptomycin (Thermo Fisher Scientific, Waltham, MA USA). All cells were cultured at 37 °C in a humidified incubator with 5% CO_2_.

EBV stable infection was performed according to the method reported by Bejarano et al.^57^. Briefly, 5 × 10^5^ CNE1 cells were added into six-well plates and cultured overnight, then infected with 100 multiplicity of infection (MOI) EBV for 2 h and decanted EBV suspension, followed by adding fresh complete medium and being cultured in an incubator at 37 °C. The cells were passaged every 2–3 days and reinoculated with fresh EBV suspension after each passage. Stably EBV infected CNE1 cells, called EB^+^, were verified in supplementary methods and used in subsequent experiments. The virus uninfected group (EB^−^) was set as the negative control.

EB^+^CNE1 cells were infected with ATR-targeting shRNA lentivirus (shATR; sc-29763-V; Santa Cruz Biotechnology, CA, USA), named EB^+^shATR. Negative control (shNC; Santa Cruz Biotechnology, CA, USA) lentivirus was infected into the EB^+^CNE1 cells, called EB^+^shNC. The cells infected with lentivirus were obtained within 2–3 weeks by treating with puromycin (Thermo Fisher Scientific) at 2 µg/mL. Then, the maintaining concentration of 1 µg/mL puromycin was chosen.

The suspended THP-1 cells of 1 × 10^6^/well were seeded in six-well plates. After 48 h induction with 100 nM phorbol-12-myristate-13-acetate, they were transformed into adherent M0 macrophages and seeded in the lower chamber of the transwell co-culture system. A total of 5 × 10^5^ CNE1 cells were seeded in the upper chamber of 0.4 μm. The following experiments were divided into four groups according to the difference between the CNE1 treatments: EB^−^, EB^+^, EB^+^shNC, and EB^+^shATR.

### Western blot (WB)

In each group, the cells were harvested and lysed in ice-cold buffer (Beyotime, Jiangsu, China). WB was performed according to the standard procedures. Primary antibodies against ATR (1:1000; sc-28901, Santa Cruz), CD68 (0.5 μg/ml; ab125212, Abcam), CD86 (1:1000; 13395-1-AP, Proteintech), CD206 (1 μg/ml; ab64693, Abcam), NF-κBp65 (1:1000; 21014, SAB), NF-κBp-p65 (1:800; 11014, SAB), c-Jun (1:1000; AF6089, Affinity), PPAR-δ (1:1000; DF7442, Affinity), JNK (1:1000; 9252S, CST), and p-JNK (1:1000; 4668S, CST) were diluted in 5% bovine serum albumin, and GAPDH (1:10000; ab181602, Abcam) was used as the internal control.

### Quantitative real-time PCR

Total RNA was extracted from NPC cells and macrophages using the TRIZOL extraction kit (Invitrogen), and then reverse transcripted into complementary DNA using TAKARA reverse transcriptase (RR047A, Japan). mRNA level of the target gene was determined by qPCR using specific primers and a SYBR green PCR kit (RR820A, TAKARA, Japan). Primer sequences are shown in Supplementary Table 1. The relative mRNA levels of ATR, HLA-DR, iNOS, TNF-α, Arg1, CCL22, and VEGF were determined compared to the GAPDH control.

### TIMER software detection

According to the TIMER software^47^ (https://cistrome.shinyapps.io/timer/), the macrophage polarization factors associated with ATR were searched. Significant different cytokines were selected for subsequent enzyme linked immunosorbent assay (ELISA) validation.

### Enzyme linked immunosorbent assay (ELISA)

The culture supernatant in the co-culture system was used for ELISA detection according to TNF-α (E0133h, EIAab, Wuhan, China), TGF-β (E0124h, EIAab, Wuhan, China) kit instructions.

### Xenograft tumor assay in nude mice

Male BALB/c nude mice of 6 week were purchased from Vital River Laboratory Animal Technology (Beijing, China) and divided into four groups randomly (*n* = 6), EB^−^, EB^+^, EB^+^shNC, and EB^+^shATR. A total of 5 × 10^6^ cells for each group in 100 μl RPMI-1640 were inoculated subcutaneously into the left axilla of the BALB/c nude mice, respectively. The tumor growth curves were determined by measuring the tumor size using vernier caliper, and tumor volumes were calculated by the formula (maximum diameter × minimum diameter^2^)/2. All mice were mercifully sacrificed at day 27. The tumors were excised, some of which were frozen in liquid nitrogen for WB or fixed in 10% formaldehyde for IHC by the Ki67 assay, and others of which were instantly characterized by the FCM. All experiments were approved and carried out according to the Guidelines for the Care and Use of Animals (Animal Monitoring and Use Committee of West China Second Hospital, Sichuan University). The study was carried out under the supervision of the Ethics Committee of West China Second Hospital of Sichuan University (2018085). The investigator was blinded to the group allocation during the experiment.

### Flow cytometry analysis (FCM)

NPC cells were co-cultured with macrophages for 48 h. Then the macrophages were trypsinized and resuspended in phosphate buffer saline to a concentration of 1 × 10^7^/ml, and dispensed into 100 μl into the flow cytometry tubes. Overall, 5 μl extracellular antibody APC antihuman CD206 (321109, Biolegend, CA, USA) was added and kept still for at least 30 min, then 5 μl intracellular antibody FITC antihuman CD68 (333805, Biolegend, CA, USA), and PE antihuman CD86 (374205, Biolegend, CA, USA) were incubated in the dark for at least 30 min after permeabilization. Then protein expression was detected by flow cytometry (Beckman Coulter, Brea, CA, USA).

The xenografts were cut and dispersed into single cells, filtered through a 300-mesh filter cloth and centrifuged. The cells were resuspended in PBS to a concentration of 1 × 10^7^/ml, and dispensed into 100 μl into the tubes. The following steps were the same as the former cell experiments.

### Statistical analysis

SPSS software (version 24.0; IBM Corporation, Armonk, NY, USA) and GraphPad Prism software (version 7; GraphPad Software, San Diego, CA, USA) were used for statistical analysis. Data were presented as the mean ± SD, and one-way analysis of variance was used to compare the means. Student’s *t* test was used for pairwise comparisons between groups. Chi-squared test was used to analyze clinical samples, and *p* < 0.05 was considered statistically significant.

## Supplementary information

Supplementary Information

Supplementary Figure Legends

Supplementary Table 1

Supplementary Figure 1

Supplementary Figure 2

Supplementary Figure 3

Supplementary Figure 4

Supplementary Figure 5

## Data Availability

All data generated or analyzed during this study are included in this published article.
