# Supplementary material for: EB virus-induced ATR activation accelerates nasopharyngeal carcinoma growth via M2-type macrophages polarization
Source: Cell Death Dis. 2020 Sep 11;11(9):742. doi: 10.1038/s41419-020-02925-9 (PMC7486933; doi:10.1038/s41419-020-02925-9)
Supplement: Supplementary file 1 — Supplementary Information [file 41419_2020_2925_MOESM1_ESM.doc]

EB virus-induced ATR activation accelerates Nasopharyngeal Carcinoma growth via M2-type Macrophages Polarization

**Supplementary Methods**

**Separate EBV**

According to the previously reported method ^[1, 2]^, B95-8 cells were continuously cultured for one month. The grown cells were gently shaken every three days and a small amount of fresh medium containing 2% to 5% fetal bovine serum was added. Separation of supernatant and cell pellet was performed by repeated freeze-thaw method combined with sonication to separate EBV from B95-8 cells, followed by filtration through a 0.22 µm filter and further purification using PEG6000 (P8250; Solarbio, Beijing, China). The viral titer was calculated as 1.0×10^9^ copies/mL using real time-PCR according to the EBV nucleic acid quantitative detection kit (Sansure Biotech, Changsha, China).

**Immunofluorescence assay**

The 12 specimens from the West China Hospital of Sichuan University were analyzed by immunofluorescence assay. In brief，5 μm frozen sections were placed at room temperature for 30 min. After rinsed with PBS, the sections were incubated with 3% hydrogen peroxide. The sections were sealed by serum for 30 min and incubated with phosphorylated-ATR (1:100; ab227851, Abcam) and CD68 (1:50; bs-1432R-AF647, Bioss) at the same time. The next day, after rinsing with PBS, the sections were incubated with Alexa Fluor 488 (4 μg/ml; A-11008, Thermo Fisher) and Alexa Fluor 594 (4 μg/ml; A-11005, Thermo Fisher) for 30 min. The sections were sealed with ProLong™ Gold Antifade Mountant with DAPI (P36931, Thermo Fisher). The sections were observed under confocal microscope.

**Electron microscopic observation of EB virus particles in CNE1 cells**

The EB^+^CNE1 cells were trypsinized and collected. 0.5% glutaraldehyde was slowly added at 4 °C and kept still for 10 min, and then centrifuged at 10000~13000 rpm for 10~15 min. After discarding the supernatant, 3% glutaraldehyde was used to fix the transmission electron microscope of West China Hospital (Chengdu, China).

**Immunofluorescence**

5×10^5^ EB^+^CNE1 cells were inoculated in six-well plate preset with sterilized coverslips for 24 h. Then the coverslips were taken out, fixed with 4% paraformaldehyde for 15 min at room temperature, permeabilized with 0.2% Triton X100 for 5min, followed by blocking in 4% FBS for 30 min. Finally, the cells were incubated with green fluorescence antibodies against EBNA2 (1:200; ab90543, Abcam) for 2 h and then with an Alexa Fluor 594 dye-conjugated secondary antibody (1:500; Thermo Fisher Scientific) for 1 h before counterstaining of the nuclei with Hoechst 33342 (0.5 µg/ml; C1029, Beyotime Biotechnology, Shanghai, China) at room temperature. The slides were covered with fluorescence mounting medium (Dako, Glostrup, Denmark) and photographed under a fluorescence microscope (Olympus, Tokyo, Japan).

**Compounds/chemicals**

ATM inhibitor compounds AZD0156 （T6770, Targetmol, Boston，MA, USA） was dissolved in 100% DMSO at 10 mg stocks, which was used for in vitro studies. AZD0156 at a concentration of 5 µM for 24 h was used for the chemotherapy experiments.

**Supplementary Results**

**p-ATR was highly expressed and the CD68 positive cells were infiltrated in NPC specimens**

The location of p-ATR and CD68 was simultaneously detected by fluorescent staining. Consistent with the histochemical results, the results showed that p-ATR was highly expressed and the CD68 positive cells were infiltrated in NPC specimens. The expression of p-ATR was weaker in the NPI group, and infiltration of CD68 positive cells was lesser (SFig. 1).

**Establishment of ATR interfered EBV-positive NPC cells**

Transmission electron microscopy showed that the nucleus was irregularly interspersed with round virus particles with the diameter of 100~200nm. Some viruses showed a circular envelope (SFig. 2A). The IF test showed that there was no expression of EBNA2 in the CNE1 nucleus before viral infection. However, EBNA2 expression was significantly enhanced after 100 MOI EBV infection (SFig. 2B), and was present in most nuclei. The results of WB and qPCR showed that the mRNA and protein expression levels of ATR in EB^+^shATR were significantly lower than those of EB^+^shNC CNE1 cells (*p＜0.05, SFig. 2C).

**Induction of THP-1 cells into M0 macrophages**

THP⁃1 cells showed a single round shape and suspended growth. After being inducted with 100 nM PMA for 48 h, THP-1 cells gradually differentiated into irregular fusiform shape and pseudopod extension, showing cell attachment (SFig. 3A). FCM showed that the expression rate of CD68 in THP-1 was 12.6%, and the expression rate after PMA induction increased to 91.8% (SFig. 3B).

**ATM inhibitor AZD0156 reduced the proportion of M1 and M2 TAMs *in vitro***

We treated EB^-^ and EB^+^ group with 5 μM AZD0156 for 24 h, respectively. The WB results showed no significant difference in the expression of CD68, while CD86 and CD206 were nealy no difference (SFig. 4).

**References**

1. Bejarano, M. T., Masucci, M. G., Klein, G. & Klein, E. T-cell-mediated inhibition of EBV-induced B-cell transformation: recognition of virus particles. *Int J Cancer* **42,** 359-364 (1988).

2. Hedrick, J. A. et al. Interation between Epstein-Barr virus and a T cell line (HSB-2) via a receptor phenotypcally distinct from complement receptor type2. *Eur J Immunol* **22,** 1123-1131 (1992).
