# Supplementary material for: EB virus-induced ATR activation accelerates nasopharyngeal carcinoma growth via M2-type macrophages polarization
Source: Cell Death Dis. 2020 Sep 11;11(9):742. doi: 10.1038/s41419-020-02925-9 (PMC7486933; doi:10.1038/s41419-020-02925-9)
Supplement: Supplementary file 2 — Supplementary Figure Legends [file 41419_2020_2925_MOESM2_ESM.doc]

EB virus-induced ATR activation accelerates Nasopharyngeal Carcinoma growth via M2-type Macrophages Polarization

**Supplementary Figure Legends**

**Supplementary Figure. 1** Specimens from NPC contained more CD68^+^ macrophage infiltration and p-ATR expression. The sections were immunofluorescent double-labeled with CD68 and p-ATR antibodies. Arrows denote CD68^+^p-ATR^+^ cells in representative images.

**Supplementary Figure. 2** Successful establishment of ATR-silencing EBV-positive NPC cells. **A.** Transmission electron microscopy showed EBV particles in the nucleus (red arrow). **B.** Immunofluorescence detection of EB virus nuclear antigen EBNA2 expression in CNE1. **C.** WB and qPCR were used to detect ATR protein and mRNA expression level.

**Supplementary Figure. 3** Successful induction of THP-1 cells into M0 macrophages by PMA. **A.** Photomicrographs of THP-1 cells and M0 macrophages (×100); **B.** FCM detection of the proportion of CD68 in THP-1 cells and M0 macrophages, respectively.

**Supplementary Figure. 4** ATM inhibitor AZD0156 reduced the proportion of M1 and M2 TAMs in vitro. WB detection of protein expression of CD68, CD86 and CD206 at 24h after co-culture.

**Supplementary Figure. 5** TAMs polarization factors, CCL17, TNF and TGF-β1, have a correlation with ATR. **A.** TIMER software detected ATR and macrophage polarization factors in HNSCC (cor and p values); **B.** Cor value difference.

**Supplementary Table 1 Primer sequence**
