# Supplementary material for: EB virus-induced ATR activation accelerates nasopharyngeal carcinoma growth via M2-type macrophages polarization
Source: Cell Death Dis. 2020 Sep 11;11(9):742. doi: 10.1038/s41419-020-02925-9 (PMC7486933; doi:10.1038/s41419-020-02925-9)
Supplement: Supplementary file 3 — Supplementary Table 1 [file 41419_2020_2925_MOESM3_ESM.doc]

**Supplementary Table 1 Primer sequence**

| **Gene Name** | **Direction** | **Sequence (5’ to 3’)** |
| --- | --- | --- |
| ATR  HLA-DR | F  R  F | GCCGTTCTCCAGGAATACAG  GAGCAACCGAGCTTGAGAGT  ACAACTACGGGGTTGTGGAG |
|  | R | GCTGCCTGGATAGAAACCAC |
| iNOS | F | TCCAAGGTATCCTGGAGCGA |
|  | R | CAGGGACGGGAACTCCTCTA |
| TNF-α | F | TCTTCTGCCTGCTGCACTTTG |
|  | R | CGAGATAGTCGGGCCGATTG |
| Arg1 | F | ACAGTCTGGCAGTTGGAAGCATC |
|  | R | GGGAGTCCCCAGGAGAATCCT |
| CCL22 | F | ATGGCTCGCCTACAGACTGCACTC |
|  | R | CACGGCAGCAGACGCTGTCTTCCA |
| VEGF | F | ATGACGAGGGCCTGGAGTGTG |
|  | R | CCTATGTGCTGGCCTTGGTGAG |
| GAPDH | F | AAGGTCGGAGTCAACGGATT |
|  | R | CTCCTGGAAGATGGTGATGG |
