# Supplementary figures and images for: EB virus-induced ATR activation accelerates nasopharyngeal carcinoma growth via M2-type macrophages polarization
Source: Cell Death Dis. 2020 Sep 11;11(9):742. doi: 10.1038/s41419-020-02925-9 (PMC7486933; doi:10.1038/s41419-020-02925-9)

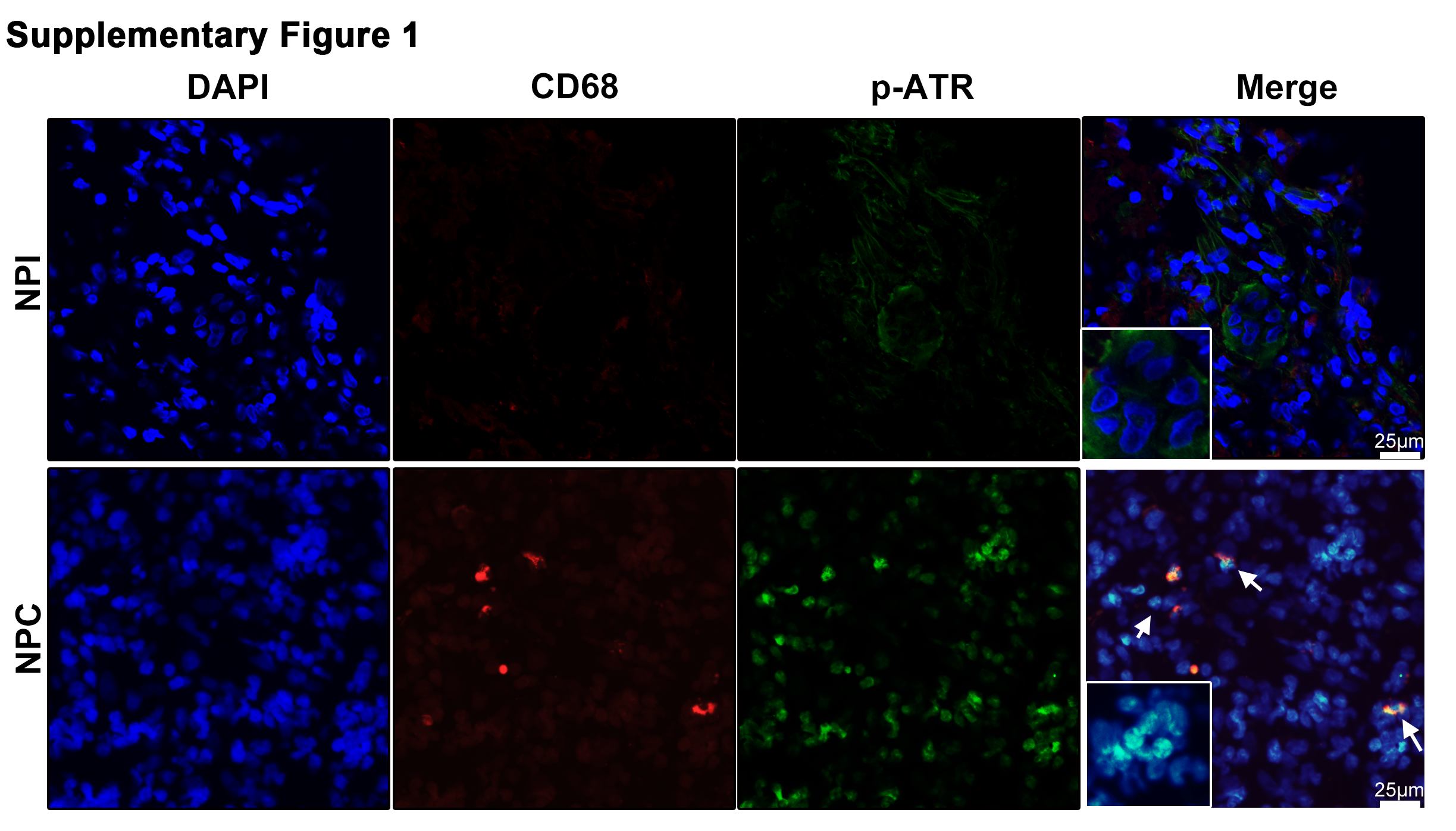

Supplement: Supplementary file 4 — Supplementary Figure 1 [file 41419_2020_2925_MOESM4_ESM.tif]

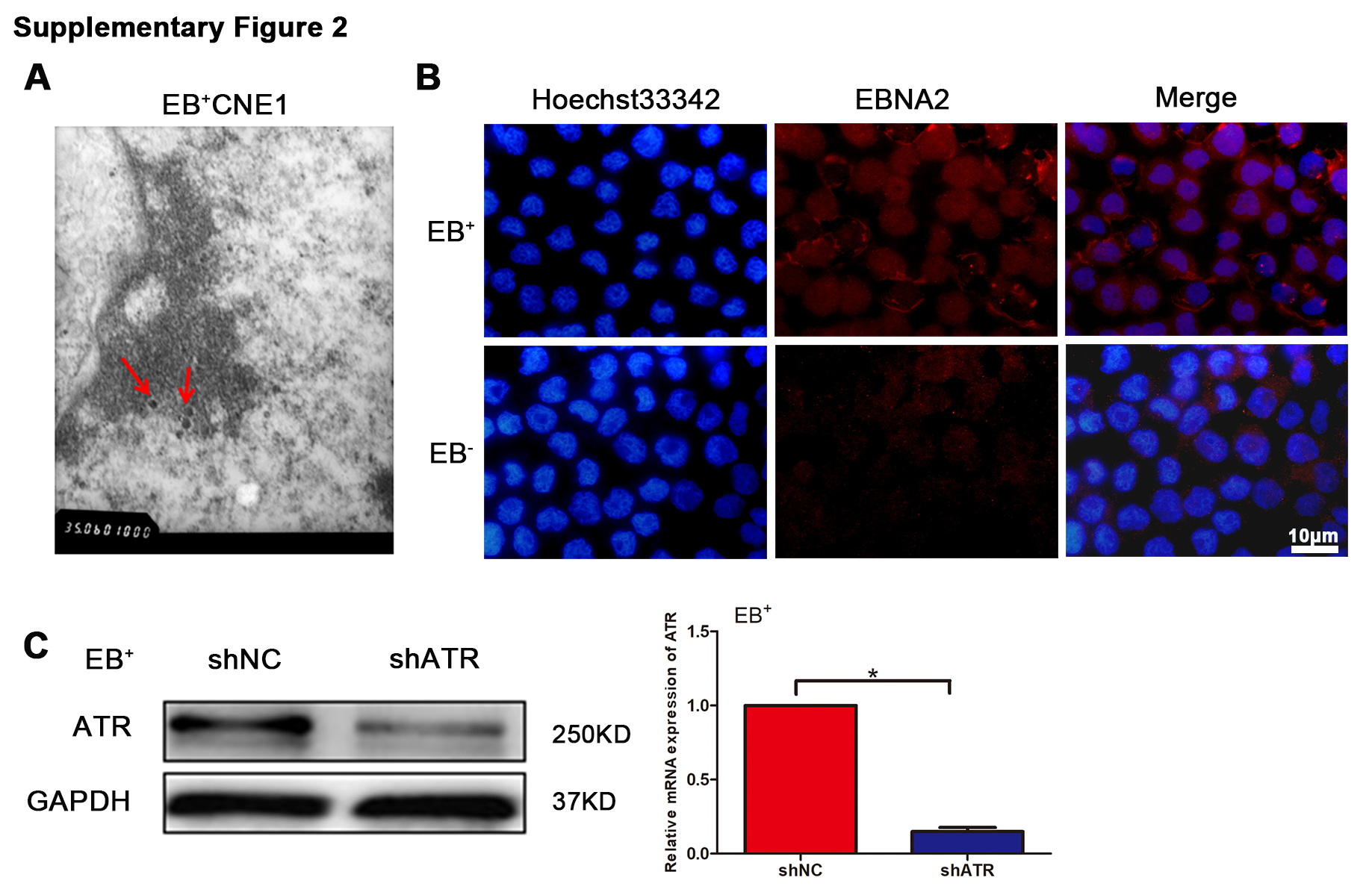

Supplement: Supplementary file 5 — Supplementary Figure 2 [file 41419_2020_2925_MOESM5_ESM.tif]

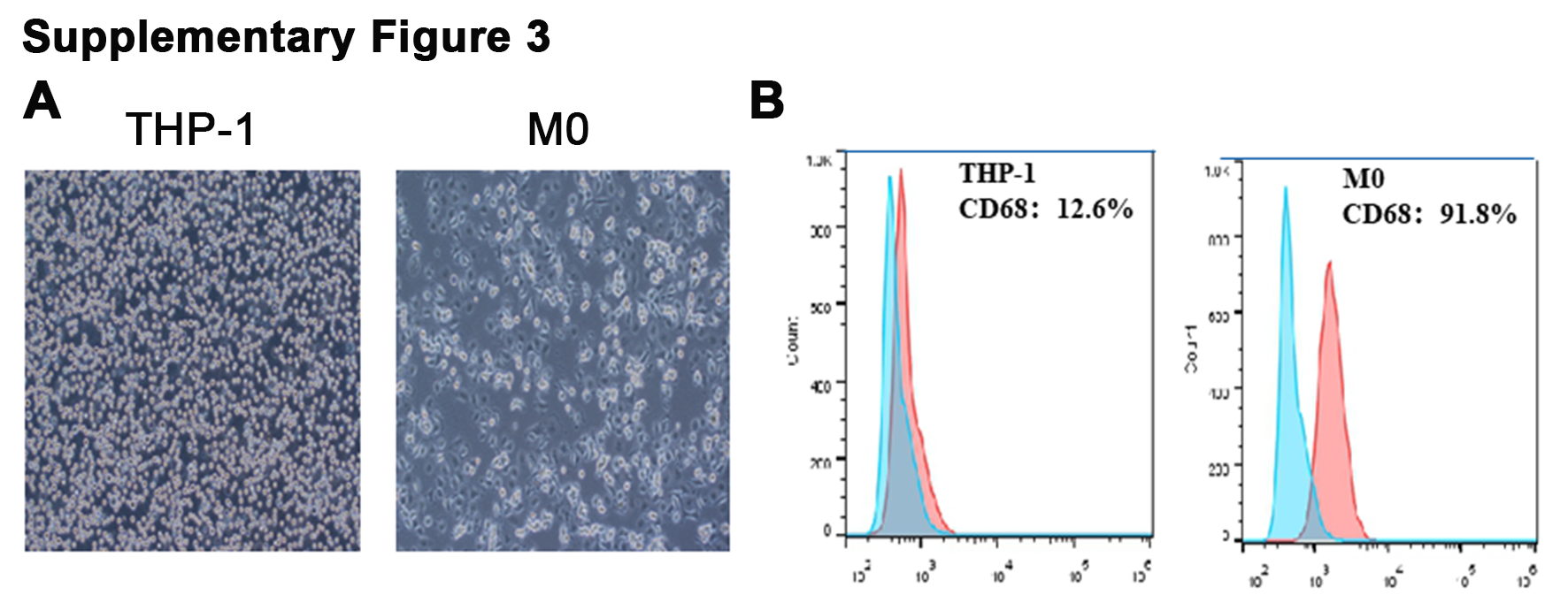

Supplement: Supplementary file 6 — Supplementary Figure 3 [file 41419_2020_2925_MOESM6_ESM.tif]

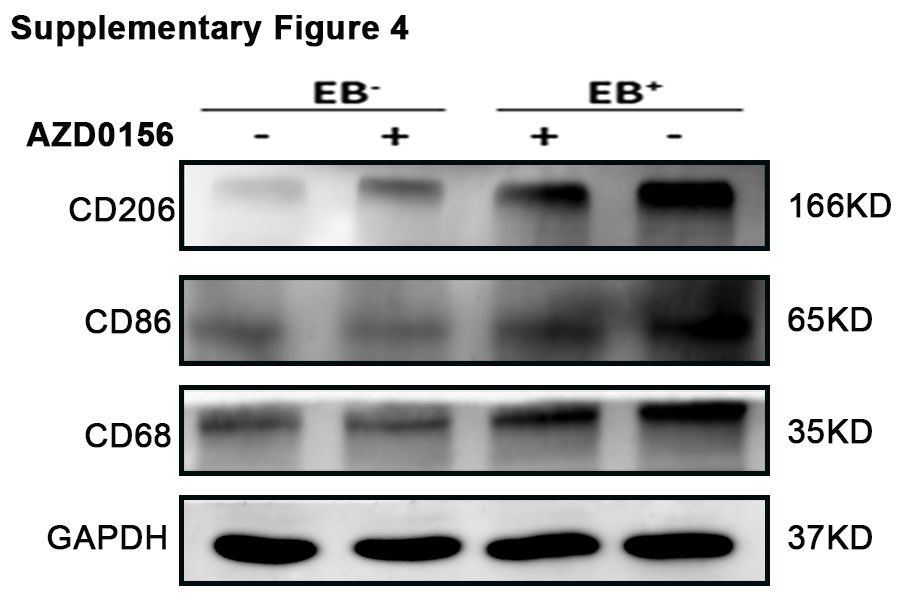

Supplement: Supplementary file 7 — Supplementary Figure 4 [file 41419_2020_2925_MOESM7_ESM.tif]

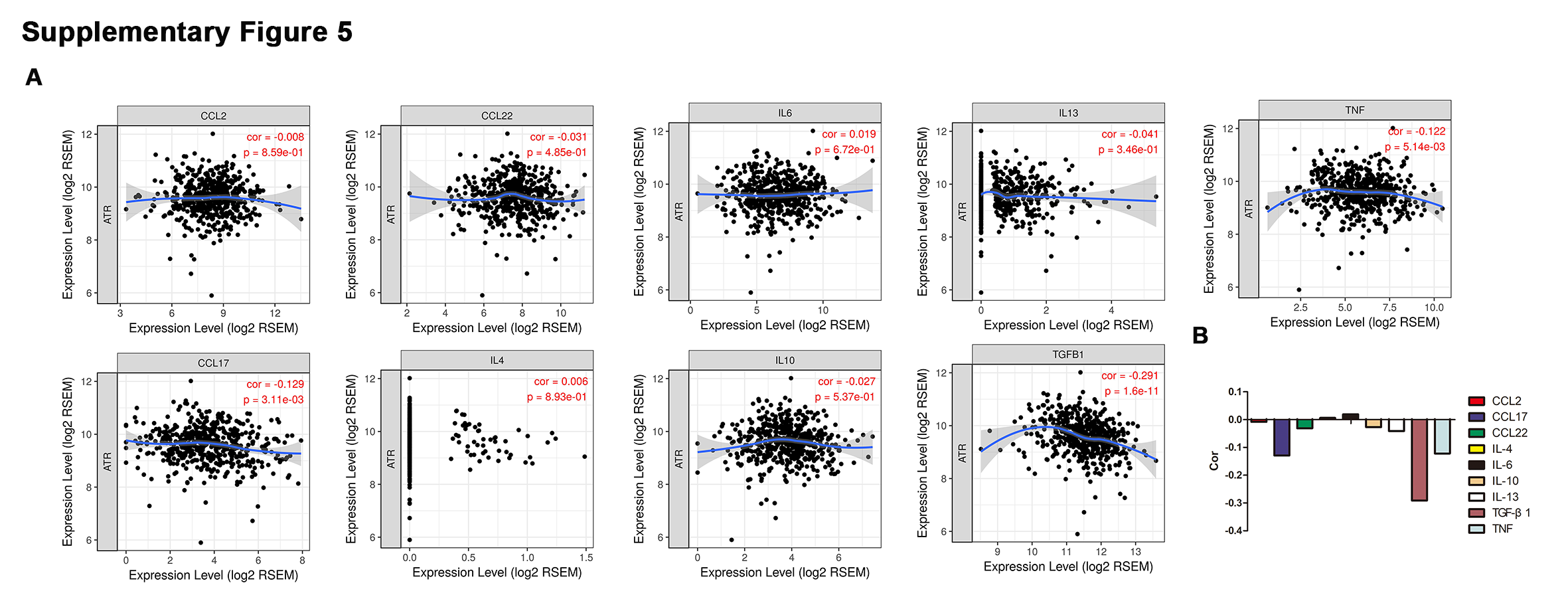

Supplement: Supplementary file 8 — Supplementary Figure 5 [file 41419_2020_2925_MOESM8_ESM.tif]
